# Supplementary material for: Three-Dimensional Large-Scale Fused Silica Microfluidic Chips Enabled by Hybrid Laser Microfabrication for Continuous-Flow UV Photochemical Synthesis
Source: Micromachines (Basel). 2022 Mar 30;13(4):543. doi: 10.3390/mi13040543 (PMC9026117; doi:10.3390/mi13040543)
Supplement: Supplementary file 1 [file micromachines-13-00543-s001.zip › micromachines-1649977-supplementary.pdf]

micromachines

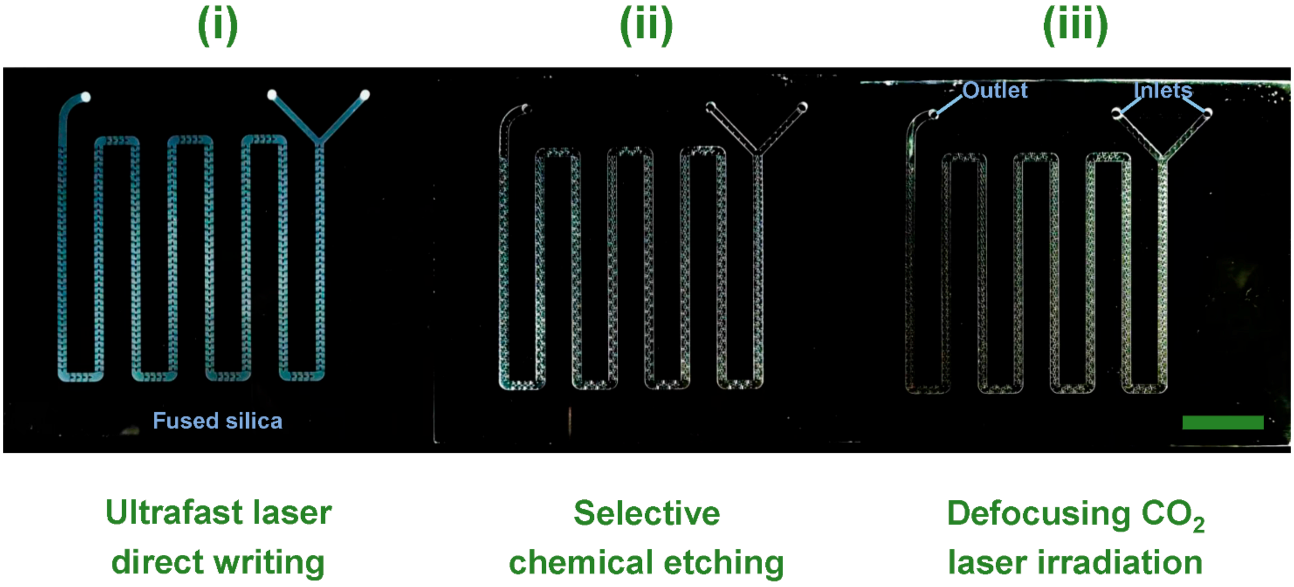

Figure S1. Photos of a fused silica glass plate with a size of 155 mm × 125 mm × 2 mm at each fabrication step as illustrated in Figure 1a. (i) Ultrafast laser direct writing; (ii) Selective chemical etching; (iii) Defocusing CO_2 laser irradiation. Scale bar represents 30 mm.

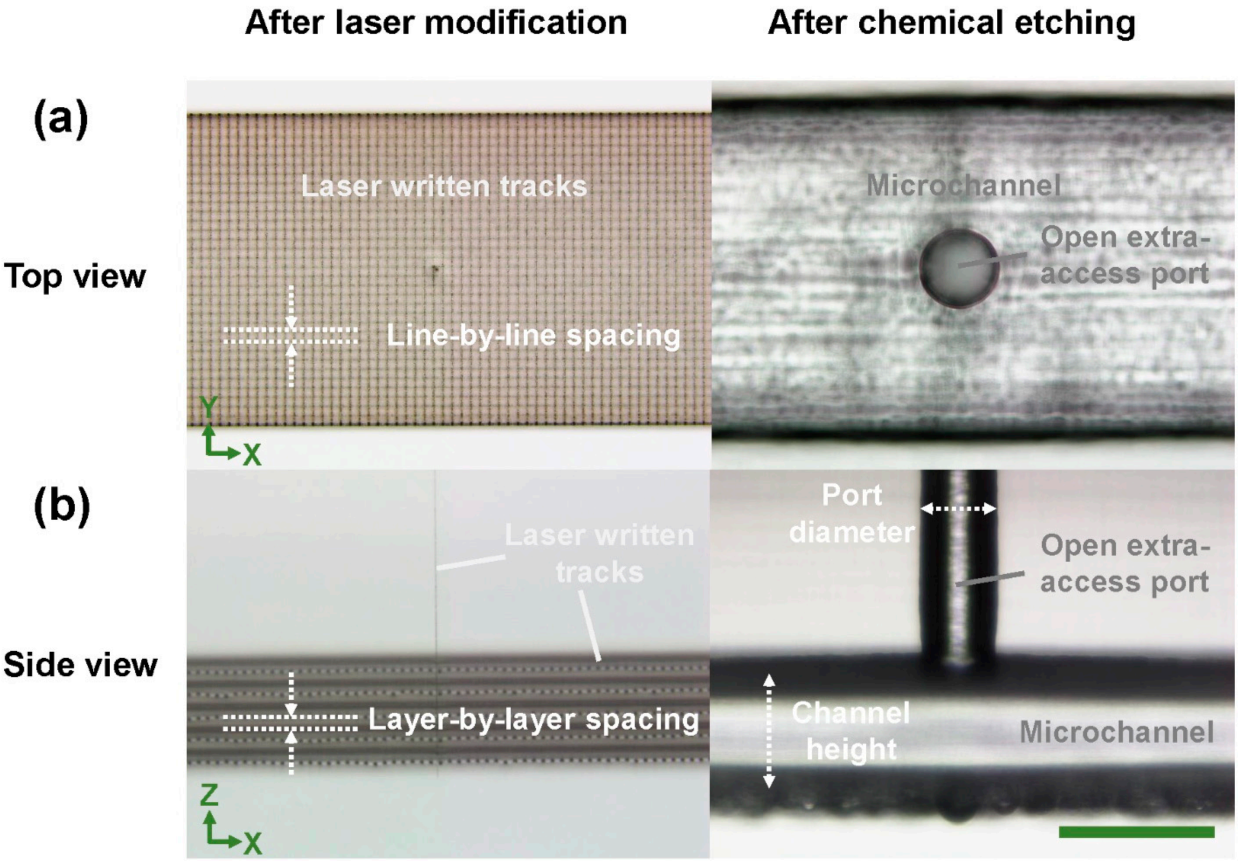

Figure S2. Top-view (**a**) and side-view (**b**) optical micrographs of a microchannel with an extra-access port after ultrafast laser modification and after chemical etching. To demonstrate the laser direct writing scheme, the schematics of line-by-line spacing and layer-by-layer spacing are indicated in (**a**) and (**b**), respectively. Scale bar represents 0.5 mm.

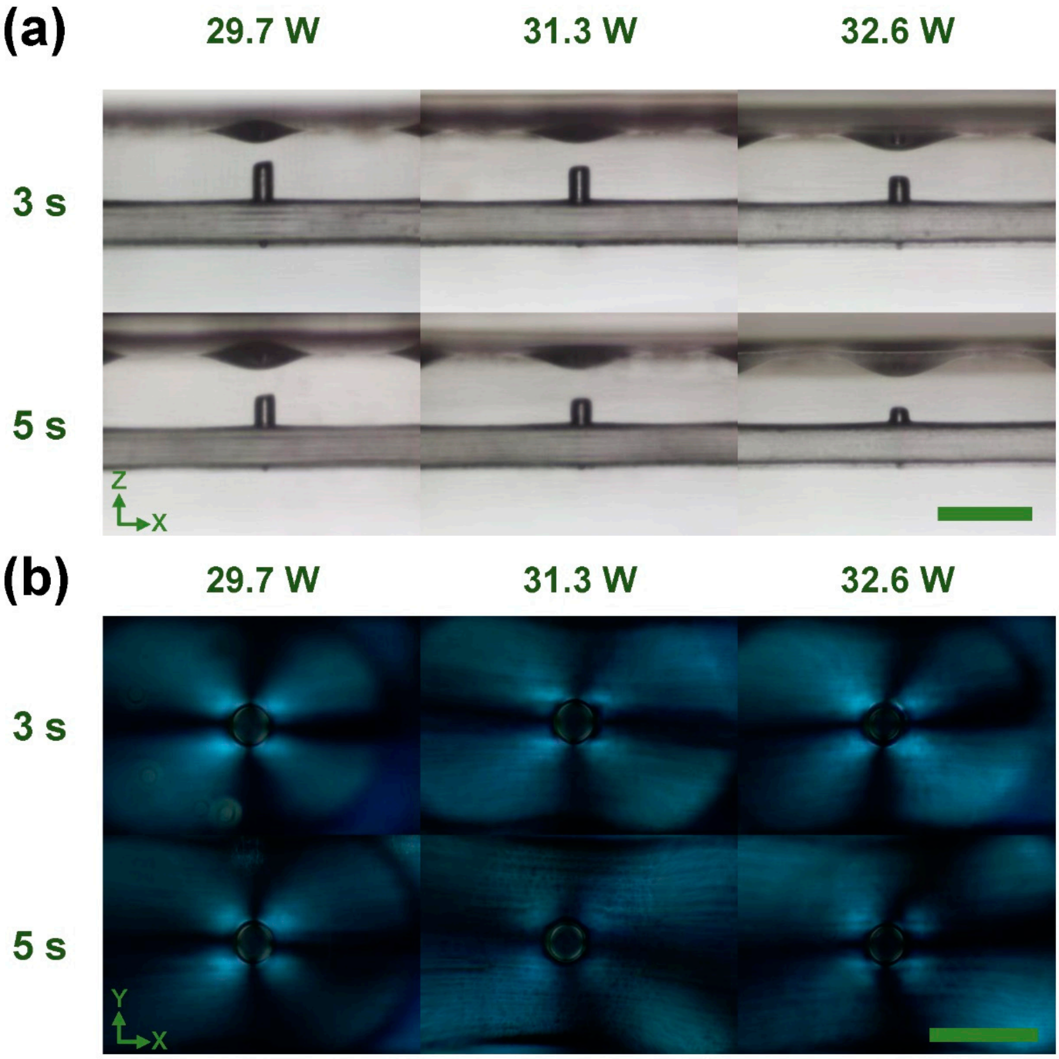

Figure S3. (**a**) Cross-sectional optical micrographs and (**b**) front-view polarized optical micrographs of a microchannel structure with an extra-access port after defocusing CO_2 laser irradiation at different laser powers (29.7, 31.3, and 32.6 W) and irradiation times (3 and 5 s). Scale bars in (**a**) and (**b**) represent 1 mm and 0.5 mm, respectively.
